# Incremental impact on malaria incidence following indoor residual spraying in a highly endemic area with high standard ITN access in Mozambique: results from a cluster‐randomized study

**DOI:** 10.1186/s12936-021-03611-7

**Published:** 2021-02-10

**Authors:** Carlos Chaccour, Rose Zulliger, Joe Wagman, Aina Casellas, Amilcar Nacima, Eldo Elobolobo, Binete Savaio, Abuchahama Saifodine, Christen Fornadel, Jason Richardson, Baltazar Candrinho, Molly Robertson, Francisco Saute

**Affiliations:** 1 ISGlobal, Hospital Clínic - Universitat de Barcelona, Barcelona, Spain; 2 Centro de Investigação em Saúde de Manhiça, Maputo, Mozambique; 3 President’s Malaria Initiative, US Centers for Disease Control and Prevention, Maputo, Mozambique; 4 PATH, Washington, DC USA; 5 PATH, Maputo, Mozambique; 6 President’s Malaria Initiative, United States Agency for International Development, Maputo, Mozambique; 7 Innovative Vector Control Consortium, Liverpool, UK

**Keywords:** Vector control, Indoor residual spraying, Insecticide‐treated nets, Insecticide resistance, Mozambique

## Abstract

**Background:**

Attaining the goal of reducing the global malaria burden is threatened by recent setbacks in maintaining the effectiveness of vector control interventions partly due to the emergence of pyrethroid resistant vectors. One potential strategy to address these setbacks could be combining indoor residual spraying (IRS) with non-pyrethroids and standard insecticide-treated nets (ITNs). This study aimed to provide evidence on the incremental epidemiological benefit of using third-generation IRS product in a highly endemic area with high ITN ownership.

**Methods:**

A cluster-randomized, open-label, parallel-arms, superiority trial was conducted in the Mopeia district in Zambezia, Mozambique from 2016 to 2018. The district had received mass distribution of alphacypermethrin ITNs two years before the trial and again mid-way. 86 clusters were defined, stratified and randomized to receive or not receive IRS with pirimiphos-methyl (Actellic®300 CS). Efficacy of adding IRS was assessed through malaria incidence in a cohort of children under five followed prospectively for two years, enhanced passive surveillance at health facilities and by community health workers, and yearly cross-sectional surveys at the peak of the transmission season.

**Findings:**

A total of 1536 children were enrolled in the cohort. Children in the IRS arm experienced 4,801 cases (incidence rate of 3,532 per 10,000 children-month at risk) versus 5,758 cases in the no-IRS arm (incidence rate of 4,297 per 10,000 children-month at risk), resulting in a crude risk reduction of 18% and an incidence risk ratio of 0.82 (95% CI 0.79–0.86, p-value < 0.001). Facility and community passive surveillance showed a malaria incidence of 278 per 10,000 person-month in the IRS group (43,974 cases over 22 months) versus 358 (95% CI 355–360) per 10,000 person-month at risk in the no-IRS group (58,030 cases over 22 months), resulting in an incidence rate ratio of 0.65 (95% CI 0.60–0.71, p < 0.001). In the 2018 survey, prevalence in children under five in the IRS arm was significantly lower than in the no-IRS arm (OR 0.54, 95% CI, 0.31–0.92, p = 0.0241).

**Conclusion:**

In a highly endemic area with high ITN access and emerging pyrethroid resistance, adding IRS with pirimiphos-methyl resulted in significant additional protection for children under five years of age.

Trial registration: ClinicalTrials.gov identifier NCT02910934, registered 22 September 2016, https://clinicaltrials.gov/ct2/show/NCT02910934?term=NCT02910934&draw=2&rank=1.

## Background

There has been remarkable success in the global fight against malaria since 2000. During the period 2000–2015, coordinated malaria control efforts helped reduce worldwide malaria mortality rates in all ages by 47%, averting an estimated 4.3 million malaria deaths [1]. This progress was particularly impressive in Africa, where infection prevalence was halved and clinical cases reduced by 40%, averting an estimated 663 million cases, during the same time period [2]. Most of this progress (81% of the cases averted) in Africa can be attributed to the successful scale-up of malaria vector control with conventional pyrethroid insecticide-treated nets (ITNs) and indoor residual spraying (IRS) [2].

Despite this overall success, recent trends indicate that maintaining high intervention coverage is challenging and that the number of malaria cases has increased slightly, but consistently, every year since 2016, this is mainly driven by a few high-burden countries [3, 4]. This interrupted progress has put the malaria fight at a crossroads [3] and threatens attaining the disease burden reduction targets set forth by the World Health Organization (WHO) in the Global Technical Strategy for Malaria 2016–2030 (GTS) [5]. Further complicating the picture for vector control [5, 6] is knowledge that the continued effectiveness of currently available tools is threatened by the spread of insecticide resistance (especially pyrethroid resistance) in key vector populations [7, 8]. Indeed, extensive modelling conducted in preparation of the GTS suggests that innovative approaches are needed to get back on track to achieving the proposed goals [9]. These needs include better access to prevention and treatment interventions, better distribution systems, better tools with non-pyrethroid insecticides, and optimized combinations of available tools.

The efficacy of ITNs to reduce malaria incidence, prevalence, and even all-cause child mortality has been well established [10, 11], and so this intervention has become the main malaria vector control method worldwide with an estimated 72% of households at risk in sub-Saharan Africa owning at least one ITN in 2017, as compared with 47% in 2010 [4]. Usage has also steadily increased; it is estimated that 50% of the population at risk in sub-Saharan Africa, including 61% of children under 5 years and 61% of pregnant women, slept under an ITN in 2017 [4].

The impact and cost-effectiveness of IRS as a malaria control intervention has also been clearly established by historical and programme documentation [12, 13]. One major challenge has been the increased cost of IRS with new insecticides or third generation IRS products (3GIRS). This increased cost of IRS products was associated with a reduction in IRS coverage throughout sub-Saharan Africa. Globally, the proportion of the population at risk protected by IRS was 5% in 2010 but declined to 3% in 2017 as countries identified insecticide resistance and new effective 3GIRS insecticides were more expensive [4]. In sub-Saharan Africa, IRS coverage experienced a marked decline from 10.1% (80 million people protected) in 2010 to 5.4% (51 million people protected) in 2016 before rising again to 6.6% (64 million people protected) in 2017 [4]. This and other intervention coverage gaps, as well as a funding plateau, have been identified as important contributors to the stall in progress seen in 2017 and 2018 [4, 14].

The use of 3GIRS, with longer residual activity, in addition to high ITN coverage is one approach that could improve vector control and enhance disease burden reduction in some situations. The current evidence for this potential benefit is mixed. Although modelling suggests additional incremental impact and observational studies suggest added value for IRS in addition to ITNs [15–18], experimental hut studies [19–21], non-randomized [22], and cluster-randomized trials [23–27] show variable impact that is highly dependent on transmission intensity, vector bionomics, ITN coverage, insecticide resistance profiles, implementation strategies, and other factors. A recent metanalysis on the combined used of IRS and long-lasting insecticidal nets (LLINs) concluded that care is needed when using the limited available evidence for policy decisions [28]. Regarding cost-effectiveness, there are important logistical costs associated with IRS, and while, a 2011 systematic review of IRS found it was cost-effective in low income setting [29], only one trial has explicitly evaluated the cost-effectiveness of the combined approach, and did so in the unique context of a low-burden region of Ethiopia [30]. Moreover, the added value and cost-effectiveness of IRS in addition to ITNs in the context of intense transmission areas are critical questions for the new “High burden to high impact” strategy developed by the WHO and RBM [31].

Although implementation is often sub-national, at least 35 countries in Africa already recommend combining ITNs and IRS[4], and the latter is often deployed in areas targeted by mass ITN distribution campaigns. Combining IRS and ITNs can result in different insecticides in the same area [32]. Indeed, the WHO guidelines for vector control [33] suggests that combined deployment can be used as part of an insecticide resistance management strategy, but specifically cautions against introducing a second intervention to compensate for deficiencies in the implementation of the first.

Robust data are needed to guide decisions about prioritizing and combining vector control strategies in the context of different transmission dynamics, changing insecticide resistance patterns, and limited funds [34]. To help address this information gap in a high-intensity transmission setting with evidence of emerging pyrethroid resistance, a cluster-randomized trial was conducted, assessing the impact of IRS with a microencapsulated formulation of the organophosphate insecticide pirimiphos-methyl (PM) on malaria transmission, compared to no IRS. Both arms received ITNs in accordance with the national distribution campaigns, which resulted in high ITN access in the IRS and no-IRS arms. This study explores the epidemiological outcomes of malaria incidence and prevalence over two years.

## Methods

The overall study concept, setting, and methods of this open-label, controlled, parallel-arm, superiority trial have been previously published [35].

### Study setting

The study occurred in rural Mopeia District (population 162,000) [36] in the Zambezia Province of Mozambique during 2016–2018. Zambezia is highly endemic for malaria, with parasite prevalence exceeding 60% and significant direct and indirect costs associated with the disease in some recent assessments [37, 38]. The main vectors were *Anopheles funestus* and *Anopheles gambiae *sensu lato (*s.l*.) and data from neighbouring districts showed pyrethroid resistance in *Anopheles gambiae s.l*. [39].

Access to ITNs was relatively high at baseline in 2016, as Mopeia District received 175,000 pyrethroid ITNs in a mass distribution campaign in 2013 (which represented more than one ITN per habitant) and benefits from routine distribution in antenatal clinics. Mopeia received IRS (with DDT and then pyrethroids) from 2007–2011 and in 2014 [40]. In Mozambique, ITN coverage is sustained through routine distribution at antenatal care clinics. In 2017, the NMCP conducted a mass ITN distribution campaign with alphacypermethrin-treated ITNs in Mopeia. Ownership among all ages in Mopeia was 54% during the 2017 cross-sectional study and 95% in 2018. Net use in Zambezia among households with at least one ITN was 89% in the 2018 Malaria Indicator Survey. The standard of care at public health facilities (testing of all fevers with a rapid diagnostic test (RDT) or microscopy and provision of treatment with artemisinin-based combination therapy to all positive cases) and from community health workers remained unaltered beyond study efforts to prevent stock outs of malaria commodities. There were 30 community health workers providing passive testing, treatment and reporting in Mopeia throughout the study period.

### Intervention

**Fig. 1:**
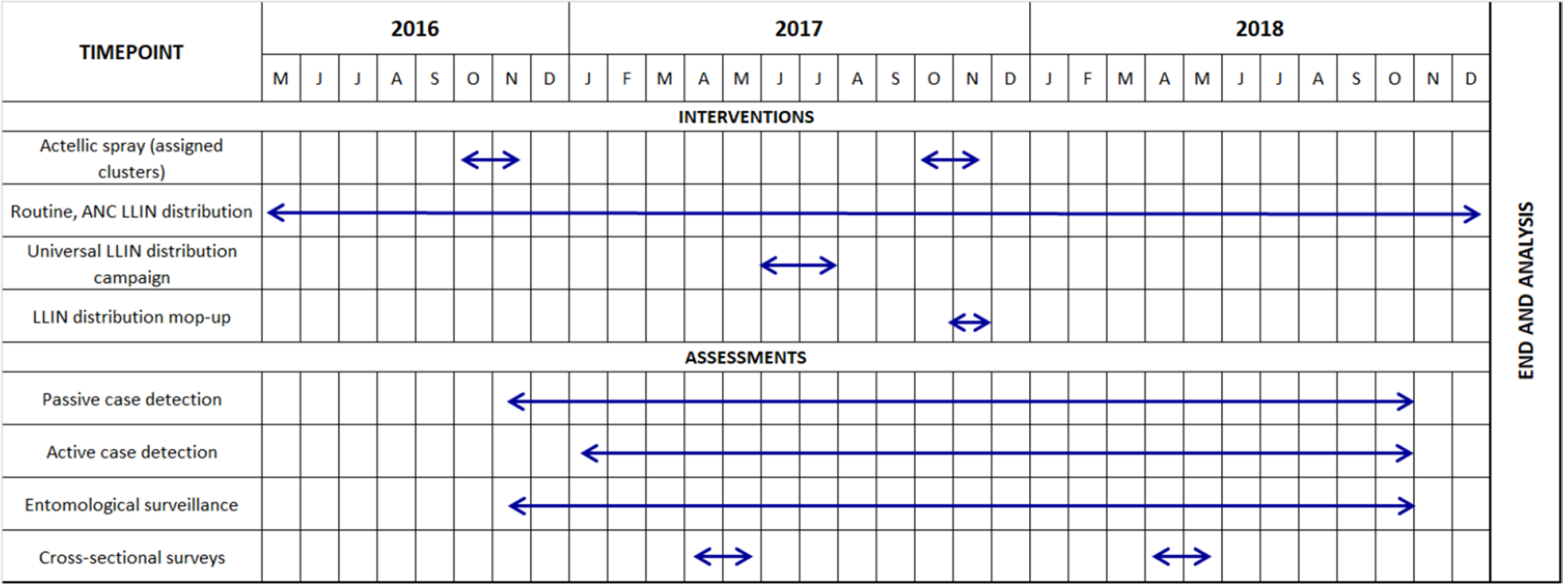
Study timeline, interventions and assessments. Considering at least nine months of efficacious indoor residual spraying (IRS) with pirimiphos-methyl (Actellic®300 CS), there was an overlap of IRS with older nets throughout 2017 and newer nets throughout 2018

Given expected impact at community level, IRS with PM was implemented only in the IRS-assigned clusters by the President’s Malaria Initiative Africa Indoor Residual Spraying (PMI AIRS) project from October–November in both 2016 and 2017 (Fig. 1). Spraying was conducted according to PMI AIRS standard operating procedures, including community and household consent.

### Study design

The study employed a two-arm, cluster randomized, controlled study design. A household and population enumeration was conducted from June–July 2016, which identified 139,286 total residents (26,320 under five years old) living in 21,328 households distributed across 194 villages. Cluster limits were delineated using expanded village borders through Voronoi polygons, with villages not reaching the minimum population for inclusion combined with the nearest neighbouring village to form a single cluster [27].

The primary research question was: In an area with high malaria endemicity and high ITN access, what is the incremental benefit of IRS with PM on reducing malaria transmission in a cohort of children under five years of age? The primary outcome was malaria infection incidence in an active cohort of children under five years of age at community level. Secondary outcomes included: (1) passively reported confirmed case incidence in all ages through the national health system, including health facilities and community health workers and, (2) malaria prevalence in all ages from annual cross-sectional surveys near the peak of the transmission season (April–May).

### Randomization and masking

The 168 clusters were stratified into three groups according to the number of households (< 69 = small; 69–125 = medium; > 125 = large), and randomized 1:1 into one of the two arms, IRS and no-IRS, by drawing lots during a public community-engagement ceremony.

### Entomological surveillance

The standard PMI AIRS Mozambique vector surveillance methods and study-specific sampling strategies have been previously described [35, 41]. In short, vector densities were monitored monthly in a subset of ten sentinel study villages (selected based on preliminary mosquito density surveys, ease of access and safety) five IRS and five no-IRS villages. In each sentinel village, overnight CDC light trap collections were conducted at eight houses for three consecutive nights every month. At one additional house per village, paired indoor-outdoor human landing collections were conducted overnight on the same three consecutive nights. Subsequent molecular analyses of the collected specimens (species confirmation, *Plasmodium* spp. infection rates, and appropriate pyrethroid resistance marker frequencies) have been reported in a separate publication [42].

Standard WHO cone wall bioassay tests were performed at a subset of five randomly selected households in each of three villages in Mopeia to assess initial spray quality and estimate the residual efficacy of PM [43]. Larval collections and subsequent insecticide resistance profiling of *An. gambiae s.l*. (2017 and 2018) and *An. funestus s.l*. (2018) using the WHO tube test bioassay also followed standard PMI AIRS Mozambique methods [41, 43].

### Primary outcome measures

#### Active cohort

 86 total clusters (43 IRS, 43 no-IRS) were selected for participation in the active cohort component of the study. Eligible households were selected from the core zones of each cluster using a fried-egg design [44] with a 1-km buffer zone at the margins of each cluster, effectively leaving a buffer of at least 2 km between spray-discordant core zones [35]. No buffers were included between clusters that had been randomized to the same study arm. Malaria infection incidence at community level was determined by enrolling a cohort of children under five years of age under parental informed consent (18 children per cluster, 774 per study arm). These children were visited monthly by a trained field worker that administered a short questionnaire to the caregiver and performed an HRP2-based rapid diagnostic test (RDT). Every child with a positive RDT received treatment with artemether-lumefantrine (AL) according to Mozambique National Malaria Control Programme guidelines at baseline and in every subsequent visit. Person-time at risk was reduced by ten days after each treatment to account for the prophylactic effect of lumefantrine [45].

#### Passive case detection

 The incidence of confirmed malaria cases (defined as fever, either reported or measured plus a positive RDT) that sought care in the public health system in Mopeia was measured using an enhanced passive surveillance approach: a study worker was placed in each of the 13 health facilities in the district to assure the quality of malaria case recording and to register the village origin of every case by village study-code.

#### Cross-sectional surveys

 A cross-sectional survey was conducted in April–May in 2017 and again in 2018 to assess malaria prevalence in all-ages and to gather behavioural information as well data on costing, and health care expenditure.

### Statistical considerations

For the active cohort, 42 clusters of 12 children per arm had 80% power at a 5% significance level to detect a reduction in baseline incidence of 30% (from estimated 700/1,000 [46] children-years to 490/1,000 children-years), using a robust *K* of 0.5. The number of clusters per arm was 43 and the number of children per cluster was 18 at enrolment to account for potential sample loss. Power and sample size calculations were conducted using the Hayes and Bennett formula [47]. The sampling strategy for each cross-sectional survey (770 individuals, half under five years of age) aimed for 5% precision to measure an estimated prevalence of 50% in a population of 128,000.

Primary analysis was done on intention-to-treat, assuming that all individuals living in an IRS cluster received IRS in their household. The effect of IRS was estimated using negative binomial regression models with the generalized estimating equations (GEE) approach. This effect was adjusted for the variables identified as potential confounders in univariate models; the interaction term between IRS and ITNs was included in the multivariate analysis. Sensitivity analyses and additional per protocol analysis adjustments were done considering ITN ownership and usage, household socioeconomic status, and cluster size (as defined by number of households). The analysis was performed using Stata Statistical Software (StataCorp 2017).

### Ethical reviews and registration

All procedures were reviewed and approved by PATH’s Research Ethics Committee, CISM’s IRB, and the National Ethics Committee of Mozambique as well as the PMI Operational Research Committee. This study was reviewed by the Centers for Disease Control and Prevention (CDC) and determined to be human subjects research with non-engagement by CDC staff. The trial was registered at clinicaltrials.gov with the identifier NCT02910934.

## Results

### Total population and study flow

**Fig. 2:**
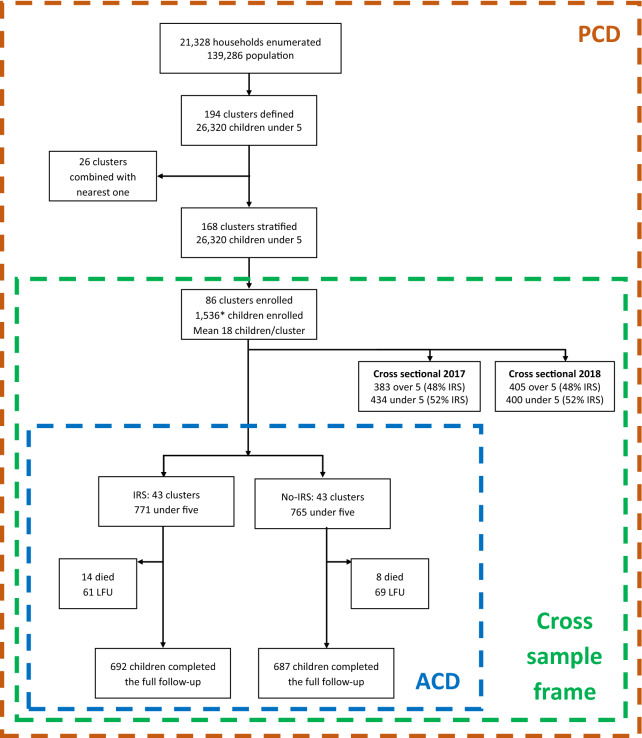
Study flow chart. ACD: active case detection, PCD: passive case detection. *of the enrolled 1,536 children, three were under six months at enrolment and 54 were between 5 and 5.5 years

The study enumeration and enrolment process are depicted in Fig. 2.

### IRS quality, acceptance and ITN distribution

The IRS campaigns were well accepted in Mopeia, with 16,500 structures (83%) sprayed of the 19,992 target in 2016 and 16,936 structures (85%) of the 19,950 target sprayed in 2017. These targets were based on PMI AIRS-led structure enumeration which was conducted pre-spray each year. Standard WHO cone bioassays using susceptible *An. gambiae *sensu stricto (*s.s*.) in houses from Mopeia district indicated that both IRS campaigns were of high quality, with all houses tested in both years showing 100% mortality within 48 h of spraying. Additionally, PM was efficacious for at least three months in 2017 [41]. In 2018, results again showed residual efficacy for a minimum of three months on all wall surface types tested, though on mud walls efficacy was of longer duration and lasted for at least four months [43].

In June–July 2017, all villages in Mopeia received 120,765 ITNs in the context of the mass distribution campaign. There were no reported stock-outs of RDTs or anti-malarials reported at health facilities in Mopeia during the study period. Following the 2017 campaign, the four-month time point measurement showed 82% mosquito mortality on mud walls of Cero village and a five-month measurement in neighbouring Mocuba and Morrumbala districts showed 95% mortality.

### Active cohort detection

Baseline characteristics were calculated using the active cohort first measurement to ensure comparability between clusters as the passive case recording did not delineate village of origin prior to the study period. A total of 1,536 children under five years of age (765 the no-IRS arm and 771 in the IRS arm), were enrolled in the active cohort from the 86 clusters (43 ITNs-only and 43 ITNs + IRS). The distribution of cluster size was equal in both groups, with 14 small, 14 medium and 15 large clusters per arm.

The baseline characteristics of the cohort are shown in Table 1. There were no major differences in terms of distance to the nearest health facility from the cluster´s centroid, ITN ownership, basic socioeconomic characteristics, age, or gender of the children enrolled. More than 60% of the children had a positive RDT at enrolment.

**Table 1:** Baseline characteristics of the children in the active cohort, Mopeia, Mozambique

	Spray Status		p-value
	No-IRS	IRS	
Cluster Characteristics (N = 86)			
Km to nearest health facility^a^	6.1 (4.7) [43]	6.8 (4.3) [43]	0.4828 ^b^
Household Characteristics (N = 1536) ^e^			
ITN ownership ^c^	470 / 765 (61.4%)	487 / 771 (63.2%)	0.4850 ^d^
Number of ITNs in the household ^a^	1.4 (0.7) [469]	1.3 (0.6) [486]	0.0868 ^b^
Electricity in the household ^c^	13 / 765 (1.7%)	8 / 771 (1.0%)	0.2641 ^d^
Head of household with any formal education ^c^	321 / 765 (42.0%)	308 / 771 (39.9%)	0.4225 ^d^
Head of household farmer c	615 / 765 (80.4%)	653 / 771 (84.7%)	0.0263 ^d^
Households enrolled (N = 1305) ^e^			
Siblings enrolled ^c^	106 / 645 (16.4%)	106 / 660 (16.1%)	0.8549 ^d^
Children enrolled (N = 1536) ^e^			
Gender: female ^c^	362 / 765 (47.3%)	389 / 771 (50.5%)	0.2193 ^d^
Age (months) at enrolment ^a^	32.4 (16.0) [765]	31.2 (16.2) [771]	0.1396 ^b^
Cluster size ^c^			
Small	243 (31· 8%)	247 (32.0%)	0.9917 ^d^
Medium	252 (32.9%)	252 (32.7%)	
Large	270 (35.3%)	272 (35.3%)	
RDT positive ^c^	474 / 765 (62.0%)	499 / 771 (64.7%)	0.2616 ^d^

^a^Arithmetic Mean (SD) [n], ^b^t-test, ^c^n (Column percentage), ^d^Chi-squared test, ^e^Note that more than one child per household could be recruited, resulting in different denominators for children in the cohort and households in the cohort

The comparison of factors potentially associated with a positive RDT between both groups at baseline is presented in the Supplementary Materials. Specifically, there were slight associations between malaria test positivity and living in a medium or large cluster, having a household sibling also testing positive, younger age, longer distances to the nearest health facility, and a history of fever in the last 48 h (Additional file 1: Table S1).

ITN ownership data collected at baseline and after the mass distribution campaign showed consistency across both arms and a large increase from 61–63% in January 2017 to 90% ownership of at least one ITN by the end of the trial (Additional file 1: Fig. S1).

**Fig. 3:**
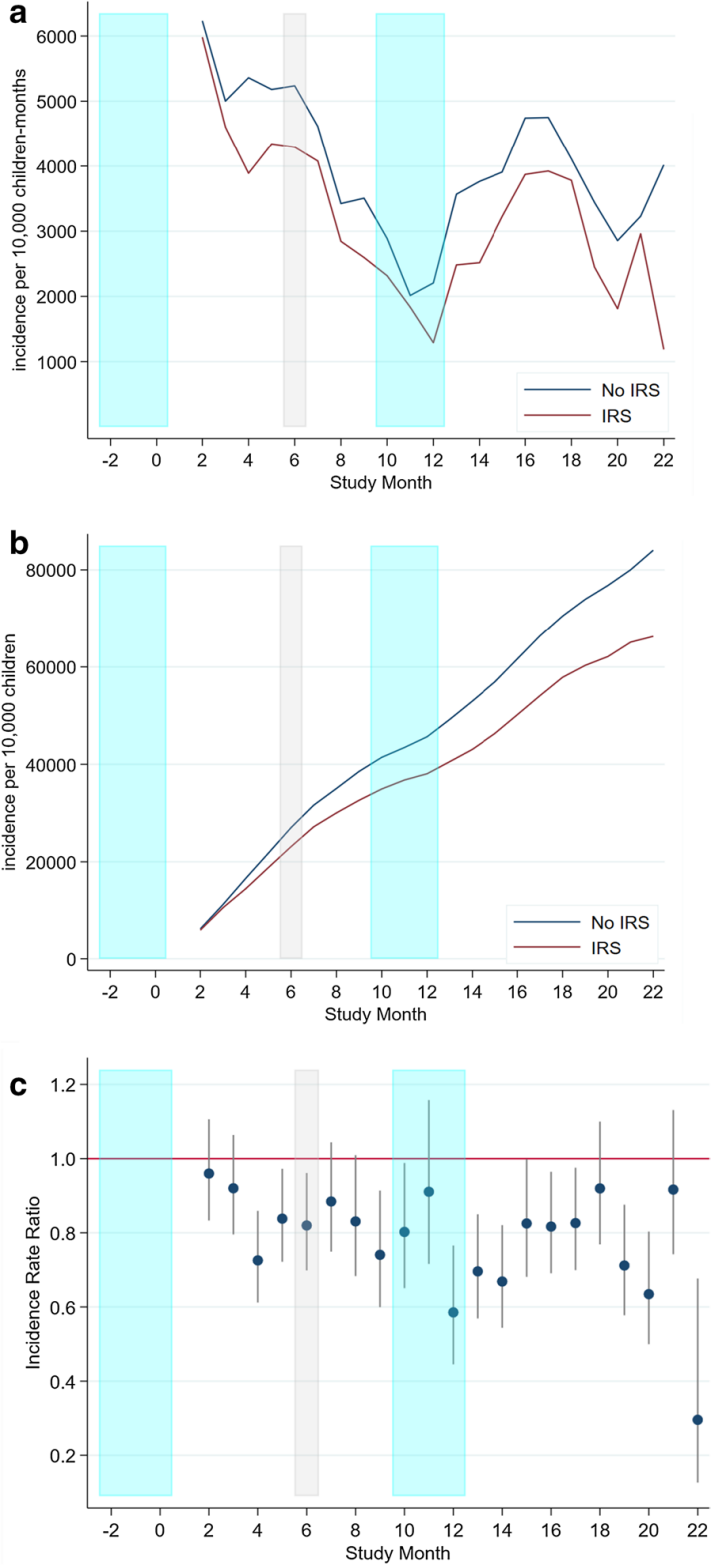
Cohort incidence by spray status (**a**); cohort cumulative incidence by spray status (**b**); and spray IRR (with 95% confidence interval) at cohort level (**c**). IRS campaigns highlighted in blue and mass ITN distribution highlighted in grey, ACT treatment correction of time at risk: 10 days

The children in the IRS arm experienced a significantly lower malaria infection incidence throughout the study. There were 4,801 cases in the IRS arm (incidence rate of 3,532 per 10,000 children-month at risk) versus 5,758 cases in the no-IRS arm (incidence rate of 4,297 per 10,000 children-month at risk). The crude risk reduction was 18% and the incidence risk ratio (IRR) was 0.82 (95% CI: 0.79, 0.86, p-value < 0.001) (Table 2 and Fig. 3).

**Table 2:** Incidence per 10,000 children-months. Time at risk corrected by ten days after each ACT treatment

Study	No IRS			IRS			Crude IRR	(95% Conf. Interval)
Month	RDT +	Cohort months at risk	Cumulative cases	RDT +	Cohort months at risk	Cumulative cases		
1	398		398	380		380		
2	374	600	772	422	705	802	0.96	(0.83, 1.11)
3	406	812	1178	353	767	1155	0.92	(0.80, 1.06)
4	345	644	1523	234	602	1389	0.73	(0.61, 0.86)
5	393	759	1916	331	762	1720	0.84	(0.72, 0.97)
6	355	678	2271	282	657	2002	0.82	(0.70, 0.96)
7	301	653	2572	282	692	2284	0.88	(0.75, 1.04)
8	227	663	2799	199	700	2483	0.83	(0.68, 1.01)
9	207	590	3006	163	628	2646	0.74	(0.60, 0.91)
10	209	724	3215	167	721	2813	0.80	(0.65, 0.99)
11	146	725	3361	137	747	2950	0.91	(0.72, 1.16)
12	154	698	3515	88	681	3038	0.59	(0.45, 0.77)
13	242	678	3757	172	693	3210	0.70	(0.57, 0.85)
14	233	619	3990	162	644	3372	0.67	(0.54, 0.82)
15	239	612	4229	204	633	3576	0.83	(0.68, 1.00)
16	326	688	4555	258	666	3834	0.82	(0.69, 0.96)
17	319	672	4874	265	675	4099	0.83	(0.70, 0.98)
18	260	632	5134	242	640	4341	0.92	(0.77, 1.10)
19	225	654	5359	160	653	4501	0.71	(0.58, 0.88)
20	187	655	5546	118	651	4619	0.63	(0.50, 0.80)
21	197	610	5743	170	574	4789	0.92	(0.74, 1.13)
22	15	37	5758	12	101	4801	0.30	(0.13, 0.68)

*IRS*indoor residual spraying,* RDT* rapid diagnostic test,* IRR* incidence rate ratio

Using these data, the IRS campaign in Mopeia averted between 15,697 and 21,651 malaria infections in the 12,670 children under five years of age living in the IRS clusters from January 2017 to October 2018. A sensitivity analysis was conducted, adjusting to account for residual HRP2 RDT positivity for up to 30 days after an infection [48], but did not show significant changes, IRR ranging from 0.79 to 0.86 (Supplementary Fig. 2).

The coefficient of variation (*k*) between clusters for RDT positive test results was calculated to be 0.336 using GEE. Given the crude incidence reduction of 18%, from 4,297 infections per 10,000 children-month (5.1 cases per child-year) in the no-IRS arm to 3,532 per 10,000 children-month (4.2 cases per child-year) in the IRS arm, the study had 74% power for its primary outcome with 43 clusters of 18 children per arm. Univariate GEE with negative binomial models were used to explore which potentially confounding factors should be included in the multivariate model. These results are shown in Table 3.

**Table 3:** Univariate analysis of covariables and their association with RDT positive status at monthly follow-up in active cohort of children under five

Variable	Crude	(95% Conf. Interval)	p-value
	IRR		
Spray Status ^a^	0.82	(0.79; 0.89)	< 0.0001
Cluster size			
Small	1.00		< 0.0001
Medium	0.95	(0.89; 1.02)	
Large	0.8	(0.75; 0.86)	
Child gender ^b^	0.95	(0.90; 1.01)	0.1077
Sibling tested positive ^c^ *(n* = *28,998, m* = *1,534)*	1.26	(1.18; 1.33)	< 0.0001
Head of household with any formal education ^c^ *(n* = *28,998, m* = *1534)*	1.04	(0.98; 1.10)	0.1752
Head of household farmer ^c^ *(n* = *28,998, m* = *1,534)*	0.98	(0.91; 1.06)	0.6856
Electricity in the household ^c^ *(n* = *28,998, m* = *1,534)*	1.05	(0.76; 1.44)	0.7696
Child with history of fever in the last 48 h ^c^ *(n* = *29,005, m* = *1,536)*	1.90	(1.83; 1.98)	< 0.0001
Participant slept under an ITN last night ^c^ *(n* = *27,479, m* = *1,521)*	0.78	(0.75; 0.81)	< 0.0001
Number of ITNs in household ^d^ *(n* = *23,175, m* = *1,535)*	0.91	(0.90; 0.92)	< 0.0001
Child age (in months) ^d^	0.99	(0.99; 0.99)	< 0.0001
Km to nearest health facility ^d^	1.01	(1.01; 1.02)	< 0.0001

n = number of observations, m = number of subjects. (n = 29,020, m = 1,536), otherwise, specified. ^a^Crude IRR for IRS vs. no-IRS cluster. ^b^Crude IRR for Female vs. Male. ^c^Crude IRR for Yes vs. No. ^d^Crude IRR per unit increase.* IRR* incidence rate ratio

Variables identified as having a significant influence on the IRR in the univariate analysis were included in a multivariate model using GEE. Table 4 shows the corrected IRR associated with IRS alone (i.e. no ITN owned), ITN use the night before, combined IRS + ITN use, having a sibling who tested positive, cluster size or distance to the nearest HF. The combined effect of sleeping under an ITN the night before in a cluster that received the IRS intervention was significantly greater than the effect of either intervention used alone: the adjusted IRR for the interaction term was 0.62 (95% CI 0.57 – 0.67; p < 0.001) corresponding to an incidence reduction of 38% (95% CI 33%-43%). The incidence reduction associated with IRS alone was 19% (95% CI 13–26%) and 23% (95% CI 18–28%) with ITN use alone (Table 4). There was a 21% risk increase in children with at least one other sibling in the cohort that tested positive. There was small but statistically significant reduction in the IRR in larger clusters and a higher risk of malaria in clusters with longer distances to health facilities (Table 4).

**Table 4:** Adjusted incidence using a multi-variable generalized estimating equation model

Variable	Adjusted	(95% Conf. Interval)	p-value
	IRR		
IRS only ^a^	0.81	(0.74; 0.87)	< 0.0001
ITN use only ^a^	0.77	(0.72; 0.82)	< 0.0001
IRS + ITN use ^a^	0.62	(0.57; 0.67)	< 0.0001
Sibling tested positive ^a^	1.21	(1.13; 1.29)	< 0.0001
Cluster size			
Small	1		0.0001
Medium	0.95	(0.89; 1.02)	
Large	0.85	(0.79; 0.92)	
Km to nearest health facility ^b^	1.01	(1.01; 1.02)	0.0001

^a^Adjusted IRR using children without ITN or IRS as referent group; ^b^Adjusted IRR per 1-km increase. Number of observations = 27,479, number of subjects = 1,521. *IRS: i*ndoor residual spraying,* ITN* insecticide treated net,* RDT* rapid diagnostic test,* IRR* incidence rate ratio

Sensitivity analyses were performed including only data after the ITN distribution campaign or adjusting the reference category for the IRR and no major changes in these results were noted (Additional file 1: Table S2).

### Passive case detection at HF

There was a total of 188 distinct villages coded during the district-wide pre-study enumeration, 81 that received IRS and 107 that did not, as per randomization which excluded a few villages that were not accessible for logistical or instability reasons. The total enumerated population was 138,685, of which 18.8% (26,097) were under the age of 5 years. Slightly less than half the total enumerated population lived in IRS villages. (68,725 = 49.6%).

There were 380,727 total visits to health facilities and to community health workers in Mopeia recorded during the study period; of these, 365,741 (96%) had a village code corresponding to a spray status with the rest corresponding to patients from outside the district boundaries, patients unwilling to disclose their home address, or visits with no village code recorded. Of visits with a corresponding village code, 174,126 (49%) included suspected malaria cases (patients presenting with, or reporting a history of, fever) that had an RDT performed: 102,004 (59%) of these RDTs were positive.

From no-IRS villages, a total of 58,030 RDT-confirmed malaria cases were recorded over 22 months, resulting in a crude all-ages case incidence rate of 361 per 10,000 person-months at risk. Almost half (48.7%) of these confirmed cases were in children under five years of age, an age-specific case incidence rate of 916 per 10,000 child-months at risk in this population. There were significantly fewer confirmed cases of malaria recorded from IRS villages: 43,973 total cases, resulting in a crude all-ages case incidence rate of 278 per 10,000 person-months at risk. Of the cases from IRS villages, 45.5% were in children under five years of age, an age-specific case incidence rate of 687 per 10,000 child-months at risk.

**Fig. 4:**
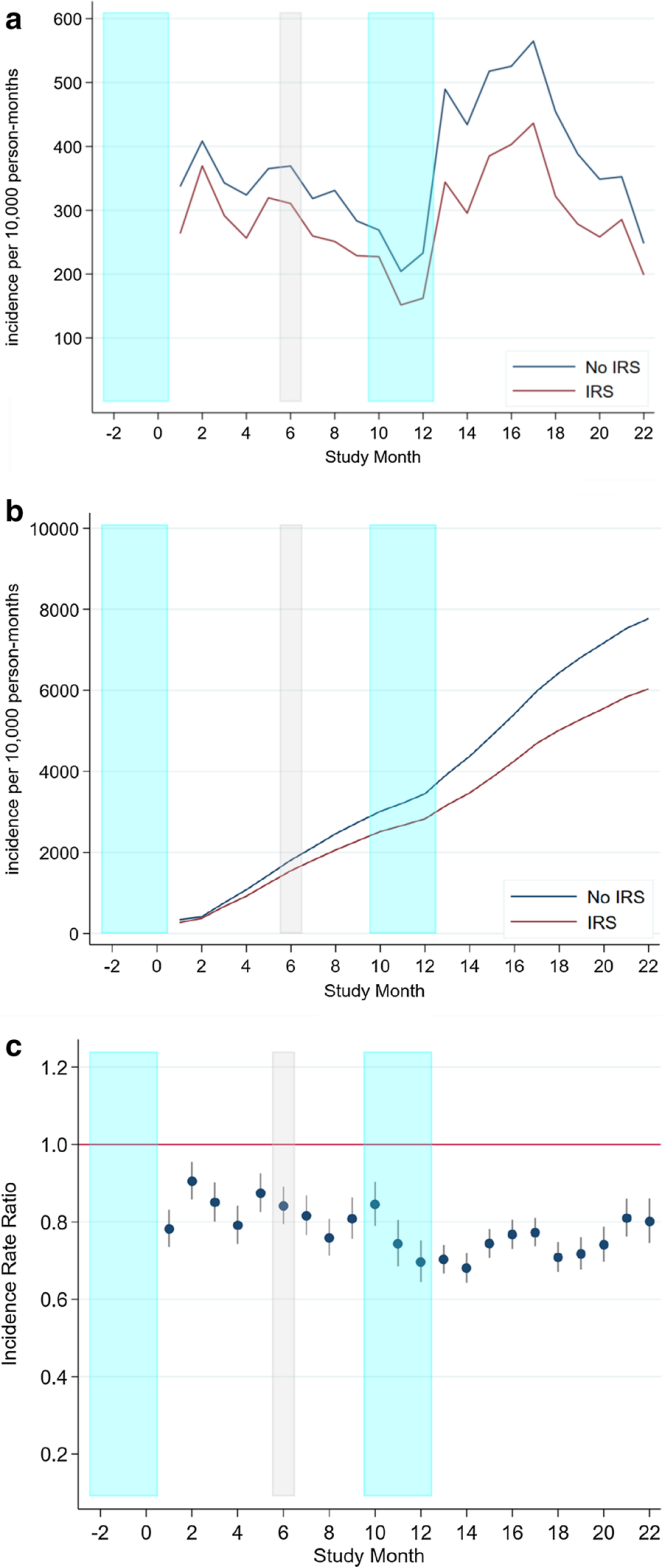
Monthly population incidence at health facilities by spray status (**a**); cumulative population incidence at health facilities by spray status (**b**); and monthly incidence rate ratio (**c**). IRS campaigns highlighted in blue and mass ITN distribution highlighted in grey

The malaria incidence at health facility in the overall population was 358 (95% CI: 355–360) per 10,000 person-month at risk in the no-IRS group (58,030 cases over 22 months) and 278 per 10,000 person-month in the IRS group (43,974 cases over 22 months), resulting in an incidence rate ratio of 0.65 (95% CI 0.60–0.71, p < 0.001). The number of averted cases was estimated to be between 15,697 and 21,651. Monthly case incidence in both arms for the overall population and children under five years of age are shown in Table 5 and Fig. 4.

**Table 5:** Malaria case incidence at health facilities in the overall and under 5 years of age population

Overall population							Under five years						
Study	No IRS		IRS		IRR	95%CI	Study	No IRS		IRS		IRR	95%CI
Month	RDT-	RDT +	RDT-	RDT +			Month	RDT-	RDT +	RDT-	RDT +		
0	740	807	677	685	0.87	(0.79, 0.97)	0	340	424	315	328	0.83	(0.72, 0.96)
1	1289	2379	1238	1811	0.78	(0.73, 0.83)	1	559	1233	523	889	0.78	(0.71, 0.85)
2	1354	2881	1323	2539	0.9	(0.86, 0.95)	2	501	1390	540	1157	0.9	(0.83, 0.97)
3	1306	2419	1381	2004	0.85	(0.80, 0.90)	3	461	1138	468	877	0.83	(0.76, 0.91)
4	1228	2286	1084	1762	0.79	(0.74, 0.84)	4	396	1098	404	747	0.73	(0.67, 0.81)
5	1353	2577	1252	2195	0.87	(0.83, 0.93)	5	442	1324	450	1023	0.83	(0.77, 0.90)
6	1332	2606	1109	2135	0.84	(0.79, 0.89)	6	450	1306	379	966	0.8	(0.73, 0.87)
7	1367	2246	1230	1785	0.82	(0.77, 0.87)	7	537	1159	487	854	0.79	(0.73, 0.87)
8	1618	2336	1408	1726	0.76	(0.71, 0.81)	8	573	1127	567	790	0.76	(0.69, 0.83)
9	1410	1999	1240	1573	0.81	(0.76, 0.86)	9	568	1019	519	786	0.83	(0.76, 0.91)
10	1449	1898	1342	1562	0.84	(0.79, 0.90)	10	555	987	506	745	0.81	(0.74, 0.90)
11	1384	1441	1196	1043	0.74	(0.69, 0.81)	11	553	673	468	458	0.73	(0.65, 0.83)
12	1306	1644	1130	1115	0.7	(0.64, 0.75)	12	508	744	471	468	0.68	(0.60, 0.76)
13	2485	3455	2014	2365	0.7	(0.67, 0.74)	13	861	1580	705	975	0.66	(0.61, 0.72)
14	2228	3063	1831	2030	0.68	(0.64, 0.72)	14	752	1353	615	784	0.62	(0.57, 0.68)
15	2471	3653	2378	2646	0.74	(0.71, 0.78)	15	808	1764	812	1165	0.71	(0.66, 0.77)
16	2189	3708	2144	2770	0.77	(0.73, 0.81)	16	823	1877	873	1313	0.75	(0.70, 0.81)
17	2111	3986	1840	2999	0.77	(0.74, 0.81)	17	702	1910	610	1289	0.73	(0.68, 0.78)
18	1961	3207	1556	2212	0.71	(0.67, 0.75)	18	668	1597	569	1078	0.73	(0.67, 0.79)
19	1971	2741	1456	1915	0.72	(0.68, 0.76)	19	660	1355	547	921	0.73	(0.67, 0.80)
20	2051	2460	1837	1775	0.74	(0.70, 0.79)	20	697	1206	725	900	0.8	(0.74, 0.88)
21	1891	2487	1696	1961	0.81	(0.76, 0.86)	21	658	1213	663	944	0.84	(0.77, 0.91)
22	1723	1751	1543	1366	0.8	(0.75, 0.86)	22	605	810	554	560	0.74	(0.67, 0.83)
**Total**	**38,217**	**58,030**	**33,905**	**43,974**	**0.78**	**(0.77, 0.79)**	**Total**	**13,677**	**28,287**	**12,770**	**20,017**	**0.76**	**(0.75, 0.78)**

IRS: indoor residual spraying, RDT: rapid diagnostic test, IRR: incidence rate ratio. The overall (under 5) population considered in the table was 68,725 (12,670) for IRS and 169,960 (13,427) for non-IRS clusters.

The crude incidence was adjusted using a negative binomial regression model with variables identified via univariate regression. These results confirmed the lower IRR in larger clusters (IRR: 0.98 per every 100 population increase 95% CI 0.98–0.99 p < 0.0001) and also revealed an increased risk of malaria detection in clusters with shorter linear distance to a health facility; people living closer to a health facility received an RDT with higher frequency (IRR: 0.68 per every 5-km increase in distance 95% CI 0.65–0.71, p < 0.0001). Data are presented in Supplementary Tables 3 and 4.

### Cross sectionals

A total of 822 participants were surveyed in 2017 and 805 in 2018. Both samples were balanced in terms of age, gender, ITN ownership, and other relevant factors (Table 6). In the 2017 survey, conducted before the mass distribution of ITNs, there was no significant difference in prevalence at the peak of the transmission season between both study arms, even when correcting by age of the participant or ITN ownership (Tables 7 and 8). In the 2018 survey, conducted ten months after ITN distribution, prevalence in children under five years of age in the IRS arm was significantly lower than in the no-IRS arm (OR 0.54, 95% CI 0.31–0.92, p = 0.0241). The incremental protective effect was particularly marked among ITN owners compared to those with no ITNs (Table 8).

**Table 6:** Characteristics of the cross-sectional samples by spray status in 2017 and 2018, Mopeia, Mozambique

	2017			2018		
	Spray Status		p-value	Spray Status		p-value
	No IRS	IRS		No IRS	IRS	
Gender Female	169 / 420 (40.2%)	174 / 397 (43.8%)	0.2986	210 / 407 (51.6%)	186 / 398 (46.7%)	0.1676
Age under 5	232 / 420 (55.2%)	202 / 397 (50.9%)	0.2123	195 / 407 (47.9%)	205 / 398 (51.5%)	0.3076
Distance to nearest HF^a^	7.1	6.8	0.7702	6.9	7.1	0.8821
ITN ownership	235 / 419 (56.1%)	204 / 397 (51.4%)	0.1783	384 / 407 (94.3%)	379 / 398 (95.2%)	0.5758
Electricity in the household	2 / 420 (0.5%)	19 / 397 (4.8%)	0.0001	2 / 407 (0.5%)	4 / 398 (1.0%)	0.4465
Head of household with any formal education	206 / 419 (49.2%)	203 / 397 (51.1%)	0.574	299 / 407 (73.5%)	292 / 398 (73.4%)	0.975
Head of household farmer	350 / 419 (83.5%)	344 / 397 (86.6%)	0.2119	368 / 407 (90.4%)	348 / 398 (87.4%)	0.1776

^a^ Mean linear distance from village centroid in km. IRS: indoor residual spraying

**Table 7:** Prevalence and odds ratio of malaria in the overall and under-five populations by spray status and age category in 2017 and 2018, Mopeia, Mozambique

	2017				2018			
	Spray Status		OR (95% CI)	p-value	Spray Status		OR (95% CI)	p-value
	No-IRS	IRS			No-IRS	IRS		
Under 5	109 / 231 (47%)	100 / 202 (50%)	1.10 (0.62,1.93)	0.7473	121 / 195 (62%)	96 / 205 (47%)	0.54 (0.31,0.92)	0.0241
Over 5	74 / 187 (40%)	71 / 195 (36%)	0.87 (0.55,1.38)	0.567	52 / 212 (25%)	40 / 193 (21%)	0.80 (0.52,1.24)	0.3241
Overall	183 / 418 (44%)	171 / 397 (43%)	0.97 (0.65,1.46)	0.8894	173 / 407 (43%)	136 / 398 (34%)	0.70 (0.49,1.00)	0.051

**Table 8:** Prevalence and odds ratio in the overall and under-five populations according to spray status and ITN ownership

	2017				2018			
	Spray Status		OR (95% CI)	p-value	Spray Status		OR (95% CI)	p-value
	No IRS	IRS			No IRS	IRS		
ITN owned ^1^	96 / 235 (41%)	94 / 204 (46%)	1.24 (0.76,2.00)	0.3869	166 / 384 (43%)	125 / 379 (33%)	0.65 (0.46,0.92)	0.0139
No ITN owned ^1^	87 / 183 (48%)	77 / 193 (40%)	0.73 (0.43,1.24)	0.2459	7 / 23 (30%)	11 / 19 (58%)	3.14 (0.80,12.32)	0.1004
Overall ^1^	183 / 418 (44%)	171 / 397 (43%)	0.97 (0.65,1.46)	0.8894	173 / 407 (43%)	136 / 398 (34%)	0.70 (0.49,1.00)	0.0514

### Entomological characterization

A full analysis of the entomological impact of the IRS campaigns will be presented in a complementary manuscript [42]. In terms of characterizing the underlying vector bionomics at the sentinel sites, more than 90% of all anophelines collected (23,974/25,735) were *An. funestus s.l.* and 97% of those tested to date by PCR have been confirmed as *An. funestus s.s.* (2,234 / 2,309) [41–43]. Samples of *An. gambiae s.l.* were also present, though in substantially lower densities (1,320/25,735; 5% of all anophelines collected, with 82% of those tested [336/411] being *Anopheles arabiensis*) [42]. Baseline, pre-intervention, dry season CDC light trap collections from September and October 2016 indicated slightly higher *An. funestus s.l.* densities at the IRS sentinel sites compared to the no-IRS sentinel sites (geometric mean 4.5 [3.5–5.8] mosquitoes per trap-night *vs.* 2.5 [1.8–3.4] mosquitoes per trap-night) [41, 42].

The WHO tube test results from 2015 showed that pyrethroid resistance was evident in *An. gambiae s.l.* populations from the nearby districts of Mocuba (52% mortality against deltamethrin/40% against lambda cyhalothrin) and Morrumbala (34% mortality against deltamethrin/33% against lambda cyhalothrin) [39], although data from Mopeia district in 2017 showed that *An. gambiae s.l.* was 100% susceptible to both alphacypermethrin and to PM. *Anopheles funestus s.l.* from Mopeia were tested in 2018 and were 100% susceptible to PM and DDT, but showed signs of emerging resistance to alphacypermethrin (85% mortality), deltamethrin (88% mortality), and bendiocarb (89% mortality) [42, 43].

## Discussion

The IRS campaigns of 2016 and 2017 made positive contributions to malaria control in this high transmission district of Mozambique, as evident by: (1) reduced infection incidence in IRS clusters relative to no-IRS clusters, even in the presence of high ITN ownership; (2) reduced confirmed clinical case incidence at public health clinics; and among those detected by community health workers, and (3) reduced odds of malaria infection in the population under five years of age during the 2018 prevalence survey.

Malaria policy makers and implementers face difficult decisions regarding the best available tools and their optimal deployment. Prior cluster-randomized trials have provided differing results, suggesting that the added value of the combination of ITNs and IRS is variable and likely.

dependent on local factors like transmission intensity, vector bionomics, insecticide resistance profiles, and implementation strategy [23–27].

This study generated robust evidence to help support those making policy and implementation decisions about the use of IRS with a non-pyrethroid insecticide in communities with high rates of malaria transmission, high ITN ownership of standard pyrethroid-only ITNs, and evidence of emerging pyrethroid resistance in the local vector populations.

This study found significant added malaria protection by adding IRS with PM to a policy of universal coverage with a pyrethroid-only ITN in Mopeia. This was quantified as 18% protective efficacy when considering new *P. falciparum* infections detected in the incidence cohort, and around 28% protective efficacy when considering confirmed cases reporting to the public health system for treatment. These suggest that when resources are available, combining these two interventions will reduce malaria incidence. Some of the reasons contributing to this incremental impact include the indoor insecticide-mosaic created by combining pyrethroid ITNs and organophosphate IRS and the benefit obtained at the household level from at least one insecticide present independently of compliance with ITN use. The adjusted analysis performed with the active cohort data confirmed that the interaction of IRS and ITNs leads to the greater incidence reduction, namely 38%. While this study provides valuable evidence on the combination of interventions, it also highlights the need to generate evidence on the value of IRS in combination with ITNs with piperonyl butoxide and non-pyrethroid ITNs to inform programmatic decision-making.

The reduced odds ratio of malaria infection observed in the population under five years of age during the 2018 prevalence survey is particularly interesting as it aligns with the protective effect of IRS also observed in the active and passive surveillance components of the study. In IRS clusters, under-five prevalence was held relatively stable from 2017 (50%) to 2018 (47%), while in no-IRS clusters under-five prevalence increased from 47 to 62%. This apparent increase in under-five prevalence in no-IRS clusters occurred in conjunction with (1) the mass ITN distribution campaign of 2017 that improved ITN access to more than 90% and (2) even as prevalence in the over-five population fell from 40 to 25% during the same time. Additionally, the combination of IRS and ITNs appeared to significantly reduce the odds of malaria infection in the under-five population by almost 50% during the five months after the second spray campaign and following the mass distribution campaign. These interesting trends highlight how complex the relationship between malaria infection incidence, malaria clinical case incidence, and malaria infection prevalence can be, particularly in very highly endemic areas with year-round transmission.

These results are in contrast from those of previous cluster-randomized trials conducted in lower transmission settings in which no benefit with a combined IRS and ITN approach showed no added benefit [23, 24, 27], but in concordance with the positive results seen in higher transmission settings [25, 32]. This raises the question of whether the incremental impact maybe dependent on the transmission level.

This study employed a robust cluster-randomized design, with a multiplicity of outcome measures, a large sample size, and close community engagement, but there are some important limitations. Children in the active cohort were subject to screen and treatment every visit, resulting in early detection of infections followed by prompt treatment, which also provided temporary prophylaxis. Given that malaria infections were more common in children in the no-IRS arm, they received proportionally more treatments (and potential prophylactic benefit) which may have reduced the difference between arms, resulting in an underestimation of the true added benefit of the combined approach as adjustments done at analysis cannot fully correct for this prophylaxis. Additionally, the net reduction in malaria incidence was lower than originally expected for sample size calculations; this was, however, partly compensated by the coefficient of variation, which, once retrospectively calculated from empirical data, was lower than assumed.

Another potential source of bias could be false-positive RDT results. The RDTs used are based on the HRP2 antigen, which can persist for several weeks after treatment potentially inflating estimates of incidence in the active cohort and adding uncertainty around the number of true infections [48]. This effect would, however, occur equally in both groups. A sensitivity analysis adjusting incidence rates by censoring positive RDT results from a second consecutive household visit showed no major difference with the main findings presented here (Supplementary Fig. 2). Both study arms also benefitted from the study team efforts to avoid stock-outs which could have contributed to lower the incidence at cohort and health facility level in both arms. Despite these potential biases, it is reassuring to note the consistency among all outcome measures with active cohort, passive surveillance and cross sectionals.

Malaria remains a challenge that will require multiple preventive as well as therapeutic intervention strategies, and many of these tools will need to be used in combination to maximize impact and reach elimination goals. Understanding when and where to combine vector control strategies requires locally relevant data to ensure that resources are invested wisely, particularly in the context of the “High burden for high impact” strategy. This study demonstrated added value for IRS with a non-pyrethroid active ingredient in the context of high coverage with standard (pyrethroid-only) ITNs, as well as good access to malaria case management commodities. This supports consideration of co-investment strategy (IRS and ITNs) in areas such as Zambezia, where transmission is high and the local primary vector species, *An. funestus s.s.*, shows moderate levels of pyrethroid resistance. The cost-effectiveness of this combined approach has been analysed in the context of this trial resulting in a separate manuscript.

## Conclusion

In 2017, Mozambique had 5% of the global share of malaria cases [4]. The results of this trial suggest consideration for the combined deployment of non-pyrethroid IRS with ITNs in areas of high transmission and emerging pyrethroid resistance. This strategy could prove especially valuable in the context of an overall increase in malaria burden and strategy put in place in an attempt to get back on track to achieving the 2030 goals as outlined in the WHO Global Technical Strategy [31].

## Supplementary Information

**Additional file 1: Table S1.** Factors potentially associated with a positive RDT at baseline by spray status.** Figure S1.** Reported net ownership in both groups throughout the trial.** Figure S2.** Cohort incidence by spray status correcting for a potential RDT residual positivity of 30 days.** Table S2.** Adjusted incidence using a multi-variable generalized estimating equation model using only data after the mass ITN distribution campaign.** Table S3.** Univariate analysis of covariables and their association with RDT-positive status at monthly follow-up at health facility. n= number of observations.

## Data Availability

The datasets generated and/or analysed during the current study are available in the Dipòsit Digital de la Universitat de Barcelona repository, http://diposit.ub.edu/dspace/handle/2445/101776.

## Abbreviations

AL: Artemether-lumefantrine AL
CDC: Centers for Disease Control and Prevention
GEE: Generalized estimating equations
GTS: Global Technical Strategy for Malaria 2016–2030
IRS: Indoor residual spraying
IRR: Incidence risk ratio
ITN: Insecticide-treated net
LLIN: Long-lasting insecticidal nets
PM: Pirimiphos-methyl
PMI AIRS: President’s Malaria Initiative Africa Indoor Residual Spraying
RDT: Rapid diagnostic test
WHO: World Health Organization
