# Supplementary material for: Incremental impact on malaria incidence following indoor residual spraying in a highly endemic area with high standard ITN access in Mozambique: results from a cluster‐randomized study
Source: Malar J. 2021 Feb 10;20:84. doi: 10.1186/s12936-021-03611-7 (PMC7877039; doi:10.1186/s12936-021-03611-7)
Supplement: Supplementary file 1 — Additional file 1: Table S1. Factors potentially associated with a positive RDT at baseline by spray status. Figure S1. Reported net ownership in both groups throughout the trial. Figure S2. Cohort incidence by spray status correcting for a potential RDT residual positivity of 30 days. Table S2. Adjusted incidence using a multi-variable generalized estimating equation model using only data after the mass ITN distribution campaign. Table S3. Univariate analysis of covariables and their association with RDT-positive status at monthly follow-up at health facility. n= number of observations. [file 12936_2021_3611_MOESM1_ESM.docx]

**Incremental impact of indoor residual spraying on malaria incidence in a highly endemic area with high access to standard insecticide-treated nets in Mozambique: results from a cluster-randomised study.**

**Supplementary material**

**Supplementary Table 1.** Factors potentially associated with a positive RDT at baseline by spray status.

| **Variable** | | **OR** | **(95% Conf. Interval)** | **p-value** |
| --- | --- | --- | --- | --- |
| Spray Status *(n = 1535)* | Non-IRS | 1 |  | 0.5520 |
|  | IRS | 1.13 | (0.76; 1.68) |  |
| Cluster size *(n = 1535)* | Small | 1 |  | 0.0154 |
|  | Medium | 1.18 | (0.71; 1.95) |  |
|  | Large | 0.62 | (0.38; 1.01) |  |
| Child gender *(n = 1535)* | Male | 1 |  | 0.6518 |
|  | Female | 0.95 | (0.74; 1.20) |  |
| Sibling tested positive *(n = 1535)* | No | 1 |  | 0.0001 |
|  | Yes | 2.07 | (1.43; 2.99) |  |
| Head of household with any formal education *(n = 1535)* | No | 1 |  | 0.9103 |
|  | Yes | 0.99 | (0.76; 1.28) |  |
| Head of household farmer *(n = 1535)* | No | 1 |  | 0.4456 |
|  | Yes | 1.15 | (0.81; 1.63) |  |
| Does your hh have: electricity *(n = 1535)* | No | 1 |  | 0.7189 |
|  | Yes | 0.77 | (0.18; 3.21) |  |
| Has the child had a fever in the last 48 hours *(n = 1533)* | No | 1 |  | 0.0192 |
|  | Yes | 1.60 | (1.08; 2.36) |  |
|  | Unk | 1.00 | - |  |
| Does your hh have any mosquito nets, and sleep under it? *(n = 1535)* | No | 1 |  | 0.4538 |
|  | Yes | 1.12 | (0.83; 1.51) |  |


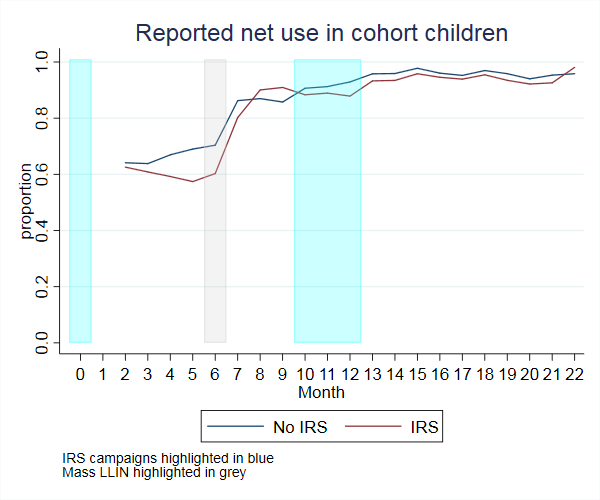


**Supplementary Figure 1.** Reported net ownership in both groups throughout the trial.


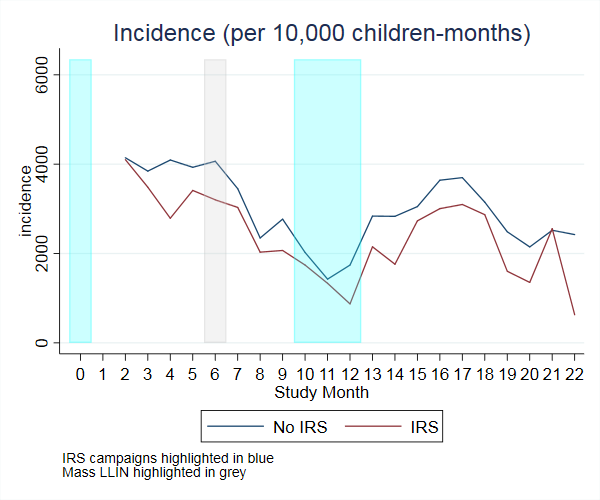


**Supplementary Figure 2.** Cohort incidence by spray status correcting for a potential RDT residual positivity of 30 days.

| **Variable** | | **Adjusted IRR** | **(95% Conf. Interval)** | **p-value** |
| --- | --- | --- | --- | --- |
| IRS only ^1^ | | 0.62 | (0.52; 0.74) | < 0.0001 |
| LLIN only ^1^ | | 0.88 | (0.78; 0.98) | 0.0257 |
| IRS + LLIN ^1^ | | 0.71 | (0.62; 0.80) | < 0.0001 |
| Sibling tested positive ^1^ | | 1.67 | (1.56; 1.79) | < 0.0001 |
| Cluster size | Small | 1 |  | 0.0020 |
|  | Medium | 0.95 | (0.87; 1.03) |  |
|  | Large | 0.85 | (0.78; 0.93) |  |
| Km to nearest health facility ^2^ | | 1.06 | (1.02; 1.10) | 0.0022 |

**Supplementary Table 2.** Adjusted incidence using a multi-variable generalized estimating equation model using only data after the mass ITN distribution campaign. ^1^Adjusted IRR for Yes vs. No, ^2^Adjusted IRR per 5 units increase. Number of obs = 19037. Number of children = 1433

|  | **Overall** | | | **Under 5** | | |
| --- | --- | --- | --- | --- | --- | --- |
| **Variable** | **Crude**  **IRR** | **(95% Conf. Interval)** | **p-value** | **Crude**  **IRR** | **(95% Conf. Interval)** | **p-value** |
| Spray Status ^1^ *(n = 3963)* | 0.72 | (0.67; 0.77) | < 0.0001 | 0.85 | (0.79; 0.91) | < 0.0001 |
| Cluster population ^2^ *(n = 3963)* | 0.98 | (0.98; 0.99) | < 0.0001 | 0.99 | (0.99; 1.00) | 0.0003 |
| Proportion of RDTs u5 ^3^ *(n = 3963)* | 0.75 | (0.55; 1.03) | 0.0713 | 2.12 | (1.44; 3.11) | 0.0001 |
| Proportion of RDTs male ^3^ *(n = 3963)* | 1.21 | (0.78; 1.89) | 0.3948 | 1.02 | (0.62; 1.67) | 0.9438 |
| Km to nearest health facility ^4^ *(n = 2316)* | 0.73 | (0.69; 0.76) | < 0.0001 | 0.66 | (0.63; 0.70) | < 0.0001 |

**Supplementary Table 3.** Univariate analysis of covariables and their association with RDT-positive status at monthly follow-up at health facility. n= number of observations. ^1^Crude IRR for IRS vs. non-IRS cluster. ^2^Crude IRR per 100 population increase. ^3^Crude IRR per unit increase. ^4^Crude IRR per five-unit increase

|  | **Overall** | | | **Under 5s** | | |
| --- | --- | --- | --- | --- | --- | --- |
| **Variable** | Adjusted IRR | (95% Conf. Interval) | p-value | Adjusted IRR | (95% Conf. Interval) | p-value |
| **Spray Status ^1^** | 0.65 | (0.60; 0.71) | < 0.0001 | 0.77 | (0.69; 0.86) | < 0.0001 |
| **Cluster population ^2^** | 0.98 | (0.98; 0.99) | < 0.0001 | 1.00 | (0.99; 1.00) | 0.2827 |
| **Km to nearest health facility ^3^** | 0.68 | (0.65; 0.71) | < 0.0001 | 0.65 | (0.61; 0.69) | < 0.0001 |

**Supplementary Table 4.** Multivariate analysis of factors associated with positive RDT at health facility. ^1^Crude IRR for IRS vs. Non-IRS, ^2^Crude IRR per 100 units increase , ^3^Crude IRR per 5-unit increase
